# Isolation and Characterization of Alpha and Nanocrystalline Cellulose from Date Palm (*Phoenix dactylifera* L.) Trunk Mesh

**DOI:** 10.3390/polym13111893

**Published:** 2021-06-07

**Authors:** Hamid M. Shaikh, Arfat Anis, Anesh Manjaly Poulose, Saeed M. Al-Zahrani, Niyaz Ahamad Madhar, Abdullah Alhamidi, Mohammad Asif Alam

**Affiliations:** 1 SABIC Polymer Research Center (SPRC), Department of Chemical Engineering, King Saud University, P.O. Box 800, Riyadh 11421, Saudi Arabia; aarfat@ksu.edu.sa (A.A.); apoulose@ksu.edu.sa (A.M.P.); szahrani@ksu.edu.sa (S.M.A.-Z.); AKFHK90@hotmail.com (A.A.); 2 Department of Physics and Astronomy, College of Sciences, King Saud University, Riyadh 11451, Saudi Arabia; nmadhar@ksu.edu.sa; 3 Center of Excellence for Research in Engineering Materials (CEREM), King Saud University, P.O. Box 800, Riyadh 11421, Saudi Arabia; moalam@ksu.edu.sa

**Keywords:** date palm trunk mesh, cellulose, lignocellulosic waste, alpha cellulose, nanocellulose

## Abstract

Highly pure cellulosic polymers obtained from waste lignocellulose offer great potential for designing novel materials in the concept of biorefinery. In this work, alpha-cellulose and nanocrystalline cellulose were isolated from the date palm trunk mesh (DPTM) through a series of physicochemical treatments. Supercritical carbon dioxide treatment was used to remove soluble extractives, and concentrated alkali pretreatment was used to eliminate the lignin portion selectively to obtain alpha-cellulose in approximately 94% yield. Further treatments of this cellulose yielded nanocrystalline cellulose. The structure–property relationship studies were carried out by characterizing the obtained polymers by various standard methods and analytical techniques such as Fourier transform infrared spectroscopy-attenuated total reflection (FTIR-ATR), thermogravimetric analysis (TGA), differential scanning calorimetry (DSC), energy dispersive X-ray diffraction (EDX-XRD), scanning electron microscopy (SEM) and transmission electron microscopy (TEM). Almost 65% yield of pure cellulose was achieved, out of which 94% is the alpha-cellulose. This cellulose shows good thermal stability and crystallinity. The microscopic analysis of the nanocellulose showed a heterogeneous mix of irregular-shaped particles with a size range of 20–60 nm. The percentage crystallinity of alpha-cellulose and nanocellulose was found to be 68.9 and 71.8, respectively. Thus, this study shows that, this DPTM-based low-cost waste biomass can be a potential source to obtain cellulose and nano-cellulose.

## 1. Introduction

The current global initiative to promote research and innovation in the area of renewable agri-resources has led to the development of novel and high-value products based on lignocellulose. For the sustainable development of non-food consumer goods and various industrial products, cellulose is a cornerstone resource available to human beings. Cellulose, a linear polymer composed of glucose units, is the most abundant of all naturally occurring organic materials, with annual production exceeding 10^11^–10^12^ tons [1]. To convert this natural polymer into advanced materials, many studies pertaining to various sources of this polymer, particularly waste agri-byproducts, different isolation strategies and its physicochemical properties have been carried out.

For many centuries, in arid regions, the date palm has been one of the most widely cultivated plants, because of its sweet edible fruits. Additionally, it has been utilized for shelter, various handicraft products and for many other applications. The Kingdom of Saudi Arabia is the second largest date producer globally. According to the 2018 statistical database of the United Nations Food and Agricultural Organization, the date production in Saudi Arabia reached 1,302,859 tonnes over 116,125 ha of harvesting areas [2]. Moreover, according to reports by Saudi Arabia’s Ministry of Environment, Water, and Agriculture, over 30 million palm trees were cultivated in 2018 [3]. As a result, substantial quantities of waste biomass are generated during the seasonal pruning and refinement of the palms. It is estimated that about 20 to 35 kg of biomass is generated per palm tree, and annually, approximately 1 million metric tons of the biomass waste is generated [4,5,6]. This vast amount of date-palm waste is either buried in landfills or burned directly in open fields, causing a severe threat to the environment. Therefore, innovative ways of valorizing this renewable resource should be analyzed for value additions [7,8]. This lignocellulosic waste biomass can be viewed as a sustainable source of cellulosic materials for various applications. Moreover, this will help address the issue of solid waste disposal to a certain extent and generate additional income sources for farmers.

Recently, a few studies have been conducted to obtain pure cellulose from selected parts of the date palm. For example, microcrystalline cellulose was extracted from date seeds by sequential treatment with alkali, and then bleaching with sodium hypochlorite, followed by acid hydrolysis. Cellulose of type I with a crystallinity index of 62% was obtained and was further enhanced to 72% after acid hydrolysis [9]. In a similar study, microcrystalline cellulose was isolated from a date palm fruit bunch stalk by treating with a bleaching agent at first, followed by alkali and then finally, subjecting it to acid hydrolysis. It was observed that cellulose fibers having diameters of 21–96 µm and lengths of more than 200 µm were obtained. Also, the cellulose so obtained was reported to have a 79.4% crystallinity index [10].

In another work, cellulose was isolated from waste date rachis, leaflets and fibers by treatment with dilute acid, alkali, followed by bleaching with acetic acid, hydrogen peroxide and sulfuric acid. A more than 70% yield of alpha-cellulose was obtained, with an average crystallinity index of 52.27% [11]. In yet another study, microfibrillated cellulose and cellulose nanowhiskers were isolated from date rachis and leaflets. Cellulose was isolated from these parts through a series of reactions, including dewaxing with two wt.% of alkali (NaOH) and bleaching with sodium chlorite(NaClO_2_). Furthermore, a high-pressure microfluidizer was used to obtain microfibrillated cellulose, while sulfuric acid treatment was used to get nanosized whiskers. Rod-shaped nanoparticles having an average diameter of 6.1 nm and length of 260 nm were also reported [12].

Besides these, very few studies have been performed to obtain nano-sized cellulosic fibers/crystals from the various parts of the date tree. Patrik Sobolčiak et al. isolated cellulose nanofibers from date leaves by treating them with 1 M ammonium persulphate solution at 60 °C with vigorous overnight stirring followed by probe sonication. Cellulose fibers with an average thickness of 18 ± 5 nm and a length of approximately 300 ± 24 nm were obtained [13]. Similarly, nanocrystalline cellulose was obtained from date tree fibers using a combination of sulfuric acid and acetic acid for hydrolysis. Cellulose nanocrystals having a good aspect ratio were obtained [14].

Similarly, Sami Boufi et al. reported the isolation of cellulose from the date rachis; first, biomass was treated at higher temperature with alkali and then bleached, followed by oxidation with 2,2,6,6-tetramethylpiperidin-1-yl) oxyl (TEMPO) in the second stage. This was followed by high-pressure homogenization to obtain cellulose nanofibers with 20–50 nm width and 200–1000 nm length. Further hydrolysis of the cellulose with high content of sulfuric acid (~65 wt.%) yielded cellulose nanocrystals with 15–25 nm average width and 150–250 nm length [15]. Likewise, date rachis was used to extract nanocellulose fibers by sequential oxidation to obtain shape-controlled fibers. Depending on the time of oxidation and specific reaction conditions, cellulose fibers with widths of 20–30 nm were obtained [16].

**Figure 1:**
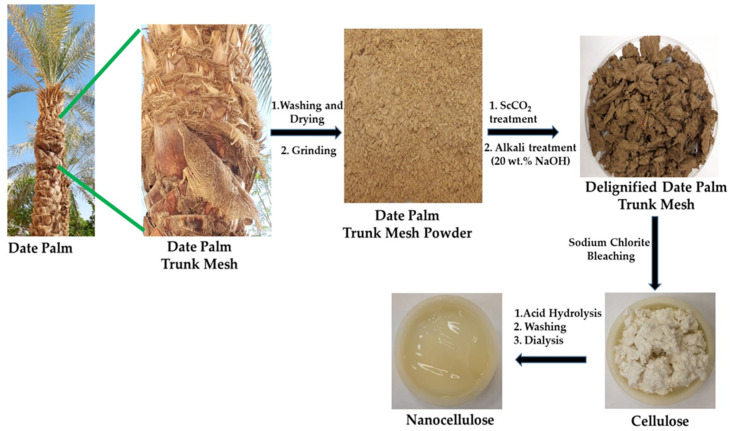
Photographs of DPTM fibers and products at various experimental stages.

In all of the previous works, a specific portion of the date tree, i.e., rachis, leaflets, seeds, etc., was used to obtain cellulose and/or nanocellulose. Comparatively, fewer studies have been carried out to obtain cellulose and/or nanocellulose from DPTM, a fibrous waste surrounding the trunk that can be obtained after cutting the fronds (Figure 1). Each date palm tree generates approximately 1.25 kg of mesh annually [17], contributing significant amounts of waste that can be valorized. Nonetheless, every feedstock remains an essential factor in extracting cellulose and other constituents under the biorefinery principle.

Over the last century, research to understand various facets of cellulose, including its isolation/extraction from various sources, gained momentum due to the increasing demand for pure cellulose and its derivatives. Additionally, according to the US Department of Energy, renewable resources will supply almost 50% of essential chemicals by 2050 [18]. Furthermore, the gross domestic product demand for nanocellulose alone is expected to increase to USD 1176 million by 2025 [19]. Recently, studies to overcome the challenges arising due to matrix–filler interaction and adhesion in the area of composites have led to an emerging concept of ‘all-cellulose composites’ [20]. In general, lignocellulosic biomass is mainly composed of cellulose, hemicellulose, lignin and, to a minor extent, pectin, waxes and inorganic minerals. The final properties and performance of the cellulose depend not only on plant locations, age of the plants, defects, and even various parts of the same plant from which it is derived, but also on several other factors such as cell dimensions, fiber structure, microfibril angles, etc. In addition, pretreatment approaches and chemicals used during extraction play a crucial role in determining the final composition of the cellulose [21].

This study focuses on the isolation and characterization of alpha- and nanocellulose from the DPTM. Alpha cellulose is a high molecular-weight portion of the pure cellulose that can withstand treatments with concentrated alkali (~17.5 wt.% of NaOH), while beta and gamma celluloses are soluble fractions [22]. For the isolation of pure cellulose, few pretreatments were carried out, including treatments with supercritical carbon dioxide, alkali and sodium chlorite. Furthermore, delignified DPTM and cellulose were characterized and used to prepare nanocellulose. The properties of the nanocellulose were thoroughly investigated for its further utilization.

## 2. Materials and Methods

### 2.1. Isolation of Cellulose

Waste fibers from a palm tree trunk were collected from the King Saud University campus. This biomass was washed several times to remove dirt, dried at room temperature and ground into fine powder. It was then treated with supercritical carbon dioxide (ScCO_2_) by using a pilot unit from PID Eng & Tech (Colmenar Viejo, Madrid, Spain). This pilot unit is composed of a L lit capacity extractor vessel that can be operated to maximum pressures up to 340 bar and a temperature up to 90 °C. Initially, the raw powder was filled into the extractor and treated with liquid CO_2_ through a pump that was maintained at a temperature below 10 °C to keep CO_2_ in the liquid state. The flow rate was kept at 1.5 L min^−1^. This pretreatment was carried out only to increase the surface area of the biomass and to remove unwanted minor components, such as waxes and other soluble extractives. The extractant was discarded without further analysis, and the solid powder was used for alkali treatments. A known amount of the above powder was treated with 20% (*w*/*v*) of NaOH solution at 90 °C for about 6 h, filtered through nylon cloth, the extractant discarded and solid materials washed thoroughly until it became neutral. It was finally dried at 100 °C and subjected to bleaching with a sodium chlorite solution using 1:0.5 weight ratio of powder to sodium chlorite. The pH of this solution was brought down to 3.7 through the addition of acetic acid, and the reaction was carried out at 70 °C for 4 h. The pure cellulose was collected by filtration, washed repeatedly until it is free from acid, and finally dried to obtain constant weight. The schematic experimental procedures and images of the products obtained are depicted in Figure 1.

### 2.2. Extraction of Nanocellulose (NC)

The extraction of cellulose nanocrystals was performed, first by twin-screw defibrillation using a procedure reported earlier [23], followed by hydrolysis with sulfuric acid. In general, mechanical treatments, such as grinding, ultrasonication, high-pressure homogenization and microfluidization, are commonly used to produce cellulose nanofibrils from the cellulosic fibers. However, these are very energy-intensive processes. Therefore, various pre- and/or post-treatment methods, such as acid hydrolysis, 2,2,6,6-tetramethylpiperidine-1-oxyl radical (TEMPO)-mediated oxidation and enzymatic hydrolysis have been explored to reduce energy consumption. This combination of chemical processes is less energy-intensive because it reduces the negative or positive charges on fiber surfaces, which in turn helps in improving the colloidal stability of the final nanocellulose [24]. On the other hand, twin-screw compounding is a very simple and continuous extrusion process and is viewed as a feasible solution for cellulose defibrillation even at nanoscale. With this method, higher yields can be achieved within a short period and at room temperature. It is estimated that the energy consumption required for this process is about 4.1 kWh/kg which is much lower than what is required in high pressure homogenization methods, i.e., approximately 30–50 kWh/kg [25].

Similarly, acid hydrolysis was carried out to convert these defibrillated fibers into nanocrystals. In this process, the amorphous or paracrystalline portion of cellulose is hydrolyzed and dissolved in acidic solution. The crystalline part, on the other hand, remains intact because it is chemically resistant to the acid. As a result, the cellulose fibrils are transversely cleaved, resulting in short cellulose nanocrystals with high crystallinities [26].

Considering all these factors, a combination of mechanical disintegration and chemical treatment with sulfuric acid was used to obtain nanocellulose in this work. For twin-screw extrusion/defibrillation, a laboratory-scale microcompounder (15 cm^3^ Xplore^®^ Sittard, Sittard, The Netherland) was used. It has a compact barrel with a conical-shaped co-rotating twin-screw and the provision of a channel for the recirculation of materials. Homogenous cellulose suspension having a solid content of ~5 wt.% was fed into the barrel and continuously recirculated within the barrel for about 30 min at a constant screw speed of 250 rpm. After this treatment, the solids were collected and subjected to sulfuric acid hydrolysis as described elsewhere [26]. In short, 10 g of treated cellulose samples were hydrolyzed in 100 mL 50 wt.% H_2_SO_4_ solution. The reaction was performed at 45 °C with continuous stirring for about 60 min. Finally, the hydrolysis reaction was quenched by the addition of a large amount of distilled water. This suspension was centrifuged several times and the supernatant fluid was discarded until it became neutral. This cloudy suspension was then dialyzed against distilled water until the pH of the suspension reached a constant value. This portion of the nanocellulose suspension was stored in a refrigerator at 4 °C; the other was freeze-dried and used for further characterization.

### 2.3. Assessment of Chemical Composition

For the determination of the chemical composition and yield, different standard methods of the Technical Association of the Pulp and Paper Industry (TAPPI) were used. Table 1 depicts the data and the detailed procedure is provided in the Supplementary Information.

### 2.4. Functional Group Analysis

Attenuated total reflectance—Fourier transform infrared spectroscopy (ATR-FTIR) analysis was carried out using a (Thermo Scientific, Winsford, UK) Nicolet iN10 FTIR microscope having a germanium microtip. The analysis was carried out in the wavenumber range of 650–4000 at a resolution of 6 cm^−1^ over 16 scans.

### 2.5. Thermal Analysis

A thermogravimetric analyzer (DTG 60H, Shimadzu., Kyoto, Japan) was used for the thermogravimetric analysis. A sample size of 10–15 mg was analyzed. The heating rate was kept at 20 °C/min with a temperature range from 30 °C to 800 °C under a continuous nitrogen flow of 50 mL/min. Similarly, differential scanning calorimetry (DSC, Shimadzu DSC-60, Kyoto, Japan) was used for thermal analysis. About 10–15 mg was sealed in the DSC aluminum pan and heating was carried out at a rate of 20 °C/min from 30 °C to 600 °C.

### 2.6. Study of Crystallinity

The crystallinities of the celluloses were analyzed using Wwde-angle X-ray diffraction (WAXRD, D8 Advance, Bruker, Berlin, Germany). An automated wide-angle goniometer coupled to a sealed-tube with Cu-Kα source radiation (λ = 1.54056 Å) was used. In reflection mode, a range of 2θ was scanned from 5° to 50° at 5°/min. Peak deconvolution was carried out using Origin software, and the Voigt function was used for the distribution of the complex pattern into the components for attaining the best fit shape. The degree of crystallinity (Xc) was assessed per the Segal method, using the following equation [26];

(1) $$CrI\left(\%\right)=\frac{I_{002}-I_{Am}}{I_{002}}\times 100$$

where, *CrI* is the percentage crystallinity index; *I*_002_ is the maximum intensity of the peak at 22°, which is a crystalline part, and *I_A_m* is the intensity of diffraction due to the amorphous part at 18°.

### 2.7. Morphological Analysis

Morphological studies were carried out at different stages of treatments by scanning electron microscopy (SEM, JSM-6360A, JEOL, Tokyo, Japan) equipped with energy-dispersive X-ray spectroscopy (EDS). The analysis was carried by placing the samples on conducting carbon tape. The accelerating voltage was kept at 6 kV for surface morphology determination, while elemental analysis by EDS was conducted at an accelerating voltage of 20 kV and with a working distance of 10 mm. All the samples were gold-sputtered by using a JEOL, JFC-1600 auto fine coater before observation to avoid samples being overcharged. The operating condition was maintained at 20 mA for 30 s. Transmission electron microscopy (TEM) imaging was performed to study the nanostructure by using a JEM-1400 (JEOL, Tokyo, Japan) field-emission electron microscope operating at an accelerating voltage of 120 kV.

## 3. Results and Discussion

### 3.1. Compositional Analysis

To obtain high-purity cellulose, effective pretreatment processes are required to remove other components, such as lignin, hemicellulose and other extractives from the recalcitrant biomass. The delignification (alkali treatment) processes removed a major amount of lignin, as seen from Table 1. The chemical functionalization of solid lignin in the alkaline delignification process is known to transform solid lignin of biomass into a soluble liquid phase [27]. Furthermore, the removal of lignin is influenced by the concentration of alkali, reaction time and temperature. It was observed that alkali treatment removed ~90% of the lignin and other soluble fractions like hemicelluloses, while bleaching with sodium chlorite removed the remainder. The maximum amount of lignin removal by alkali treatment was also reported by N Y Abu Thabit et al [9]. The reduction in kappa number indicates a lack of lignin in the biomass and can also be correlated with the amount and purity of the cellulosic components.

Similarly, alpha-cellulose represents undegraded higher-molecular-weight cellulose as mentioned earlier. The alpha-cellulose content in pure cellulose rose from 33% to 94% when compared to the DPTM. This is the highest quantity of alpha-cellulose obtained so far and is further used to make high-quality nanocellulose materials.

Moreover, according to EDX analysis (details in Supplementary Information), the raw biomass primarily contained carbon and oxygen elements with traces of inorganic elements, such as magnesium, silica, calcium, iron and chlorine. In general, lignocellulose biomass contains more than half of its compounds in the form of carbon. However, the individual components of biomass, such as cellulose, hemicellulose and lignin, have different carbon contents and properties. Lignin alone has almost 60% carbon content, followed by cellulose (42%) and hemicellulose (40%) [28]. As pristine biomass contains all of these components, including lignin, it contributes to an increase in the carbon content of the raw biomass. Furthermore, some inorganic carbons from carbonates and other inorganic sources may contribute to the increase in total carbon content in the raw biomass, as evidenced by the EDX analysis.

During alkali treatment, these inorganic elements were extracted, due to which traces of sodium could be found in the samples treated with sodium hydroxide. The cellulose and nanocellulose samples were mostly composed of carbon and oxygen, suggesting the high purity of the obtained cellulosic materials.

### 3.2. Functional Group Analysis by ATR-FTIR

**Figure 2:**
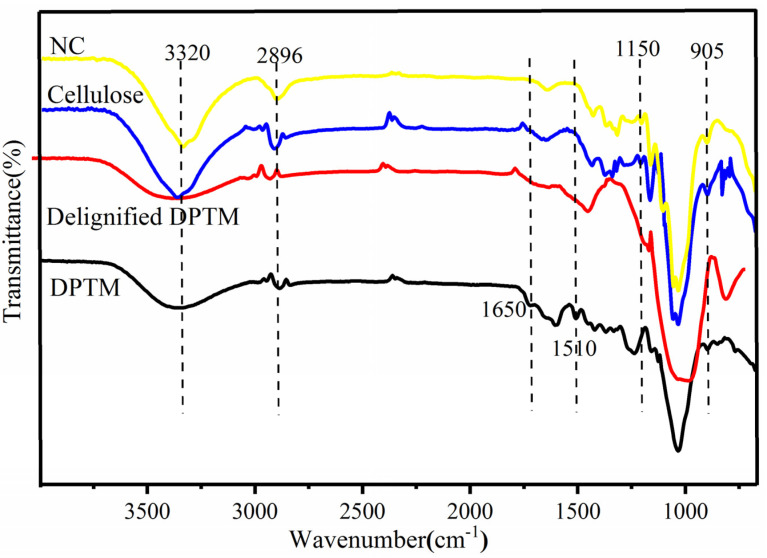
FTIR spectra of DPTM, delignified DPTM, cellulose and nanocellulose samples.

The FTIR spectra of all the samples are presented in Figure 2. A broad peak at around 3320 cm^−1^ due to stretching vibration of the O-H bond was observed in all the samples, while peaks due to C-H bond stretching frequency were detected at 2896 cm^−1^ [29]. Owing to the increased cellulose content and decreased lignin and hemicellulose portions of the raw biomass, these peaks appeared sharper for these samples. A diffused peak at 1640 cm^−1^ was due to the adsorbed moisture and indicated cellulose–water interaction [30].

Similarly, peaks at 1650 cm^−1^ and 1510 cm^−1^ indicated the presence of lignin in the DPTM sample. These are due to the stretching frequencies of C=C present in the aromatic structure of lignin and that of C=O present in the ester groups of the hemicellulose. These peaks disappeared in the spectra of the other samples, suggesting the complete removal of the lignin and thereby indicating the purity of the cellulose. In cellulose samples, the C-H vibration of the glycosidic bond was observed at 1365 cm^−1^ and 1150 cm^−1^, while the peak due to C-O-C glycosidic bond vibration (pyranose C-O-C ring stretching) appeared at 1060 cm^−1^ [31]. The peak at 905 cm^−1^ was due to C-H rocking vibrations and could be correlated to the purity of the cellulose structure [32].

### 3.3. Thermal Analysis

**Figure 3:**
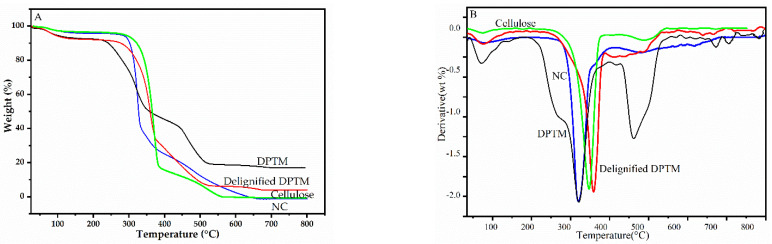
Thermogravimetric curve (**A**) and derivative thermogravimetric curve (**B**) of DPTM, delignified DPTM, cellulose and nanocellulose samples.

Figure 3 represents the relative thermogravimetric curves of the samples and Table 2 summarized the results of the analysis. The decomposition or stability can be evaluated by determining the temperature at the onset of decomposition.

The first small decomposition curve (Figure 3B) at around 100 °C represents the elimination of adsorbed water or humidity, which is nearly 5.49%, 6.20%, 2.71% and 3.5% for DPTM, alkali-treated DPTM, cellulose and nanocellulose respectively. Then samples displayed thermal stability with negligible mass loss that depends on the nature of the samples. For example, raw DPTM showed the onset of decomposition at about 221 °C, while for alkali-treated samples it was at 240 °C. This difference could be attributed to the presence of different amounts of hemicellulose (a mixture of low-molecular-weight pentose and hexose sugars) and lignin. The cellulose sample showed a relatively high onset-of-degradation temperature, 280 °C. This was found to be more than the onset-of-degradation temperature range of cellulose (150–210 °C) obtained from bleached eucalyptus pulp [33]. Similarly, it is in close agreement with the onset-of-degradation temperature of cellulose obtained from viscose wood pulp, which is reported to be 272 °C [34]. The thermal stability of cellulose depends upon its degree of crystallinity and the type and/or source of cellulose [35]. Moreover, degradation is believed to take place initially through the cellulose activation process owing, to the scission of glycosidic bonds that leads to the reduction in the degree of polymerization without losing mass [36]. This activated cellulose then releases gases like CO_2_, CO and H_2_O, which cause the partial crosslinking of cellulose, which finally contributes to the formation of solid char or tar [37].

The onset-of-degradation temperature of nanocellulose was 292 °C, considerably higher than that of cellulose. The onset-of-degradation temperature ranges for cellulose nanofibers were reported to be 184–207 °C by Y. Peng et al., 2013 [38]. According to the DTG curve, the maximum thermal decomposition of these samples occurred until 500 °C. The DPTM exhibited a typical lignocellulosic pattern with two distinct thermal decomposition phases. The first thermal decomposition occurred between 195 °C and 420 °C, corresponding to the hemicellulose and cellulose portions. Hemicellulose degrades at around 265 °C, while cellulose decomposes at temperatures ranging from 320 °C to 370 °C. The mass loss beyond 417 °C was due to the decomposition of lignin and biochar components, which took place over a wide temperature range, up to 800 °C [39].

**Figure 4:**
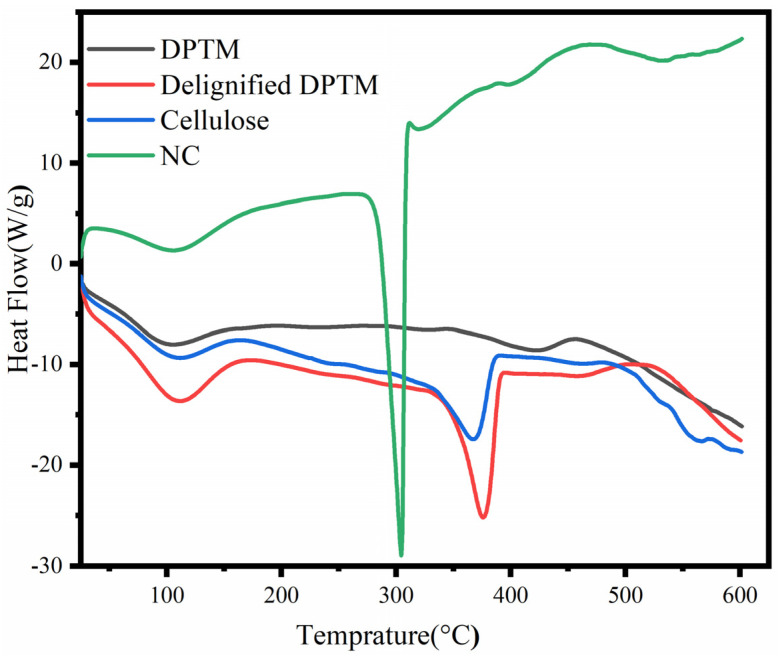
DSC curve of DPTM, delignified mesh, cellulose and nanocellulose samples.

Figure 4 shows a DSC analysis of the samples, performed to evaluate the energy consumption properties of these samples. The first broad endothermic peak was observed in the range of 80–140 °C for all samples, due to the evaporation of moisture and other volatile components. Two broad peaks appeared at 323.78 °C and 420 °C during the analysis of the DPTM sample, which corresponded to the presence of the cellulosic component and lignin, respectively [30]. The disappearance of these peaks in all other samples and the increase in the intensity of the peak due to cellulose suggested the purity of the sample. The second endothermic peak of cellulose appeared in the temperature range of 370–375 °C which could be due to the depolymerization of cellulose and decarboxylation reactions [40]. The sharp intensities of these peaks could be attributed to uniform heat absorption by the pure cellulosic components [41]. Furthermore, as a result of various treatment methods, cellulose crystallites can undergo reorientation and rearrangement, leading to a more compact crystal structure and hence, improved thermal stability [42].

An endothermic peak occurred much earlier in the case of nanocellulose due to the formation of sulfate groups (-OSO_3_H), formed due to the reaction of sulfuric acid with the hydroxyl groups during hydrolysis. This acid hydrolysis also decreased the amorphous regions, molecular weight and degree of polymerization of cellulose.

### 3.4. Wide-Angle X-ray Diffraction (WAXRD) Analysis

**Figure 5:**
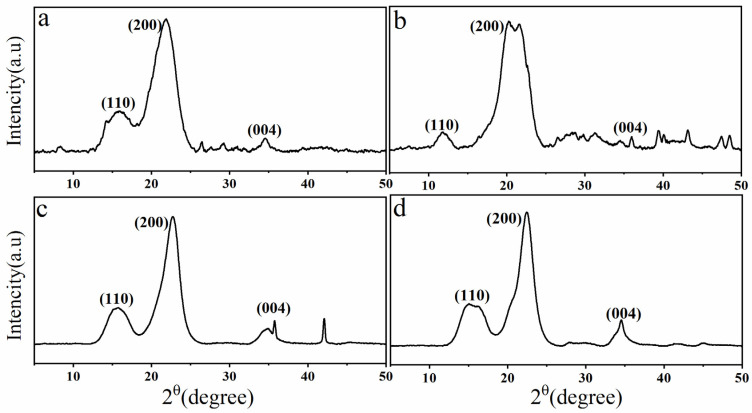
XRD diffractograms of (**a**) DPTM, (**b**) delignified DPTM, (**c**) cellulose and (**d**) NC.

Figure 5 displays XRD spectra of all the samples. As can be seen in diffractograms, in all the samples, peaks were found to be superimposed as a wide halo, due to the presence of an amorphous phase. In semicrystalline lignocellulosic polymers from softwood and vegetable fibers, this type of pattern is commonly observed [43].

Moreover, the main peaks of lignocellulosic materials were observed at 2Ɵ = 15.9°, 21.8° and 34.4° for each sample, corresponding to the (110), (002) and (004) crystal planes, respectively. This also indicated the presence of Iβ cellulose structures in cellulose and cellulose nanoparticles [44]. The sharper peak at 22.7° and 22.4° in cellulose and nanocellulose, respectively, confirmed the formation of highly ordered crystalline cellulose domains. The more prominent peaks reflected in these samples could be due to the increased crystallinity s because of the improved molecular hydrogen bonding [45]. In alkali-treated samples, the doublet peaks were observed at 20.3° and 21.5° exhibiting two types of polymorphs of cellulose, i.e., cellulose I and cellulose II. Alkali treatment by sodium hydroxide is known to partially convert cellulose I into cellulose II [46]. However, this polymorph again rearranged into cellulose I structure in the cellulose and nanocellulose after bleaching and acid hydrolysis treatments. The degree of crystallinity was estimated to be 51.3% for the DPTM samples and 56.5% for fibers treated with alkali. This is significantly more than reported by Abdal-hay et al. [47]. Additionally, an increase in the crystallinity of pure cellulose was also observed, and it was found to be approximately 68.06%. This increase in crystallinity could be attributed to the decrease in the amorphous domain due to the cleavage of glycosidic bonds present in the disordered paracrystalline section. Moreover, a bleaching reaction eventually leads to the removal of lignin and the rearrangement of cellulose molecules into more crystalline order [48]. It is also in close agreement with the crystallinity of the cellulose obtained from different parts of date fibers [11]. Similarly, the crystallinity of nanocellulose was found to be 89.61% which was higher than that of nanocellulose obtained with an oxidizing agent and homogenization treatment of palm biomass [16]. It was also found to be greater than that of nanocellulose obtained from other wood-based bioresidues such as cotton waste and oil palm leaves via sulfuric acid treatments [49,50].

### 3.5. Morphological Analysis

**Figure 6:**
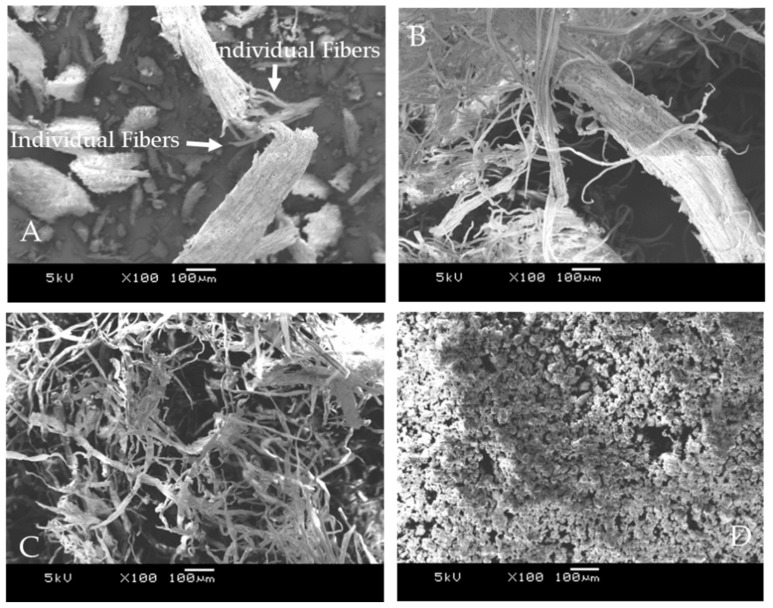
SEM images of (**A**) DPTM, (**B**) delignified DPTM, (**C**) cellulose and (**D**) NC.

In general, lignocellulosic biomass is composed of three layers of the cell wall in which semi-crystalline cellulose is reinforced with lignin and hemicellulose fractions. Morphological analysis showed that the raw date fiber bundle (Figure 6A) had a rigid structure, as evidenced by broken thick filaments. The individual cellulose fibers were packed together and displayed a compact and inflexible structure. This rigidity could arise in the raw biomass due to the rigid nature of the lignin and other cementitious components that bind these polymeric components (cellulose, hemicellulose and lignin) together as a composite structure. Significant changes to the surface of the fibers could be seen after alkali treatment with partial cellulose microfibril separation (Figure 6B). Alkali treatment is known to eliminate lignin and hemicellulose from biomass as these components are soluble in hot aqueous NaOH solution, while the cellulose fraction remains intact [51]. This treatment also removes the inorganic impurities and waxes and reduces the size of the cellulose fibers/fibrils, which eventually results in increasing the surface area of the fibers [52].

The surface of the cellulose fiber became more uniform and smooth after bleaching treatment, owing to the complete removal of the non-cellulosic components (Figure 6C). The complete defibrillation of the microfibrils into the individual fibrils resulted in a substantial reduction of the diameter of the fibrils. After exposure to air, the nanoparticles tend to agglomerate due to strong hydrogen bonding between surface hydroxyl groups of the cellulose. It is difficult to separate these fibrils (Figure 6D). Moreover, EDX analysis confirmed that the cellulose and nanocellulose are free from alkali or other residual elements and thereby also confirmed the purity of the samples (Supplementary Information).

### 3.6. Characterization of Nanocellulose

**Figure 7:**
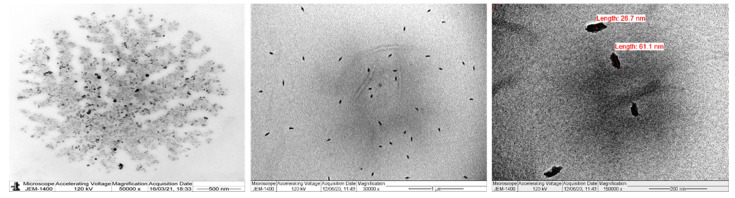
TEM images of Nanocellulose.

From Table 1, it can be seen that the acid hydrolysis of the cellulose had a negligible effect on increasing the alpha-cellulose content of nanocellulose. Cellulose chain scission via acid hydrolysis yielded nearly 89.6% of the nanocellulose. This hydrolysis also decreased the particle size of nanocellulose while further smoothing the surface morphology (Figure 5D) by eliminating the amorphous content. The TEM analysis of the particles in Figure 7 showed that the nanoparticles were agglomerated, and the native characteristics of cellulose were retained. Irregular-shaped nanoparticles with sizes ranging from 26 nm to 61 nm were observed.

Individual nanoparticles and smaller nanocrystallites were formed as a result of a reduction in hydrogen bonding caused by cellulose chain fragmentation [53]. The EDX analysis indicated that the carbon content of the nanocellulose was slightly higher than that of the cellulose, while oxygen content was found to be lower, owing to the applied hydrolysis conditions. Furthermore, the aqueous suspension of nanocellulose had a high negative zeta potential value of −35 mV. This could be due to the repulsive forces arising from anionic sulfate moieties, which contribute to the formation of a stable dispersion system. However, further research is required to determine the overall efficiency of the isolation method in terms of yield and qualities of nanocellulose.

## 4. Conclusions

In conclusion, a series of pretreatments were carried out to obtain pure cellulosic components from the waste mesh of the date palm trunk, and their structure–property relationship was analyzed in detail. The isolated celluloses exhibited similar characteristics to those reported in the literature and were free of lignin and hemicellulose components. Nearly 94% of pure alpha-cellulose, with a total yield of about 66% compared to the starting material, was obtained from this waste feedstock. The low Kappa number suggested its purity, which was also confirmed by FTIR and DSC analysis. These celluloses showed high thermal stability with onset-of-degradation temperatures of ~280 and 290 °C for the cellulose and nanocellulose, respectively. XRD analysis showed that this cellulose had the cellulose I type of polymorph, having a crystallinity of around 68% for the cellulose and 89% for the nanocellulose. According to morphological studies, the resulting cellulose had fibers with smooth surfaces, whereas nanocellulose had irregular-shaped rod-like structures with nanoscale features. Cellulose and nanocellulose are widely used in food, pharmaceutical, paint and fillers for polymer composites. Therefore, this waste agricultural biomass can be valorized as a potential source for these materials and used in such applications.

## Figures and Tables

**Table 1:** Chemical composition of date palm trunk mesh (DPTM).

Samples	α-Cellulose %	Kappa No.	Lignin %	Moisture %	Ash%	Yield %	Crystallinity %
DPTM	33.70 (±3.17)	82.06 (±3.93)	22.53 (±2.91)	6.40 (±1.01)	4.26 (±1.20)	-	51.33 (±2.06)
Delignified DPTM	65.91 (±2.50)	54.36 (±1.96)	2.16 (±0.30)	5.16 (±0.65)	2.33 (±0.51)	71.76 (±2.32)	57.66 (±2.15)
Cellulose	94.50 (±1.70)	0.39 (±0.06)	0.32 (±0.08)	4.45 (±0.50)	0.23 (±0.07)	66.53 (±3.27)	68.60 (±1.68)
Nanocellulose (NC)	92.26 (±1.35)	0.25 (±0.03)	0.13 (±0.05)	4.20 (±0.30)	-	35.26 (±4.28)	89.61 (±1.13)

**Table 2:** Summarized results of TGA analysis of samples.

Sample	T_onset_ (°C)	T_10_ (°C)	T _max_ (°C)	Residue (W%)
DPTM	221.16	241.80	321.56 (Cellulose) 461.10 (Lignin)	17.65
Delignified DPTM	240.98	275.66	358.72	6.02
Cellulose	280.50	321.09	368.50	0.62
NC	292.58	302.28	319.00	0.01

## Data Availability

The data presented in this study are available on request from the corresponding author.

## Supplementary Materials

The following are available online at https://www.mdpi.com/article/10.3390/polym13111893/s1, Figure S1: EXD analysis of raw date palm mesh, Figure S2: EXD analysis of delignified mesh, Figure S3: EXD analysis of cellulose, Figure S4: EXD analysis of nanocellulose.
