# Supplementary material for: Isolation and Characterization of Alpha and Nanocrystalline Cellulose from Date Palm (Phoenix dactylifera L.) Trunk Mesh
Source: Polymers (Basel). 2021 Jun 7;13(11):1893. doi: 10.3390/polym13111893 (PMC8200950; doi:10.3390/polym13111893)
Supplement: Supplementary file 1 [file polymers-13-01893-s001.zip › polymers-1228900-supplementary.pdf]

Supplementary information

1. **Determination of Alpha-, beta- and gamma-cellulose (Adapted from TAPPIT 203 om-99 method)**

In general, the alpha-cellulose indicates undegraded higher-molecular-weight cellulose content in pulp; beta-cellulose indicates that of undegraded cellulose, and the gamma-cellulose consists mainly of hemicellulose.

This method for the determination of alpha-, beta- and gamma-cellulose can be applied to bleached or dignified samples. Cellulose extracted consecutively with 17.5% and 9.45% sodium hydroxide solutions at 25°C. The soluble fraction, consisting of beta and gamma-celluloses, is determined volumetrically by oxidation with potassium dichromate, and the alpha-cellulose, as an insoluble fraction, is derived by difference

I. 0.5 gm of the oven-dried sample was taken in 100 ml capacity of the beaker, and 100 ml of 17.5% NaOH was added. The temperature of the reaction was adjusted to 25°C.

II. The sample was stirred till it completely dispersed.

III. After 30 minutes from the addition of 17.5% NaOH, 100 ml of distilled water was added and reaction mixture stirred for another 30 minutes at 250°C.

IV. After the completion of 60 minutes, the suspension was centrifuged. Filtrate and residues and were collected.

α-cellulose determination

I. In 250 ml capacity of a conical flask, 25ml of the above filtrate was taken with the help of a pipette, and to it was added 20 ml of 0.5 N of potassium dichromate solution.

II. To the above mixture, 50 ml of concentrate H_2SO_4 was added dropwise.

III. This solution was allowed to remain hot for 15 minutes, and then 50 ml of distilled water was added so that the reaction mixture comes to room temperature.

IV. To this solution, 2-4 drops of ferroin indicator were added and titrated against 0.1 N ferrous ammonium sulfate solutions until the mixture's red colour was obtained.

V. Blanks titration was made by substituting filtrate with 12.5 ml of 17.5 % NaOH and 12.5 ml of distilled water.

$$\alpha - Cellulose\ \% = \frac{100 - 6.85(V2 - V1) \times N \times 20}{A \times W} \qquad Equation\ 1$$

Where,

V1= titration of filtrate, ml

V2= blank titration

N = Exact normality of FAS solution

A =Volume of filtrate used in the oxidation, ml

W=Weight of the oven-dry sample, gm

β- and γ-cellulose determination

I. 50 ml of filtrate was taken with the help of a pipette into a 1000 ml graduated cylinder, and to it, 50 ml of 3N H2SO4 was added.

II. This cylinder was then kept in a water bath and heated to 70-90°C for few minutes to coagulate the β-cellulose.

III. The precipitate was allowed to settle down overnight and then centrifuged to obtain a clear solution.

IV. To 50 ml of this clear solution, 10 ml of 0.5N $K_2Cr_2O_7$ solution and then 90 ml of conc. H_2SO_4 was added portion-wise.

V. This solution was allowed to remain hot for 15 minutes, and then 50 ml of distilled water was added so that the reaction mixture comes to room temperature.

VI. To this solution, 2-4 drops of ferroin indicator were added and titrated against 0.1 N ferrous ammonium sulfate solutions until the mixture's red colour was obtained.

VII. Blanks titration was made by substituting filtrate with 12.5 ml of 17.5 % NaOH and 12.5 ml of distilled water, and 25 ml of 3 N H2SO4.

Calculations,

$$\gamma - Cellulose\ \% = \frac{[6.85(V4 - V3) \times N \times 20}{25 \times W} \qquad Equation\ 2$$

Where,

V3= titration of solution after precipitation of β-cellulose, ml

V4= blank titration

$$\boldsymbol{\beta - Cellulose}\ \% = \mathbf{100} - (\boldsymbol{\alpha - cellulose}, \% + \boldsymbol{\gamma - cellulose}, \%$$

2. Kappa number determination of delignified cellulose (Adapted from TAPPI-T 236 om-99 method)

The Kappa number is the number of millilitres of tenth-normal potassium permanganate solution consumed per gram of moisture-free pulp under conditions specified in this standard. The results are corrected to be equivalent to a 50% consumption of the permanganate in contact with the specimen. This method is generally used to determine the relative hardness, bleachability, or degree of delignification of pulp.

Relationship with lignin: The kappa number gives a straight-line relationship with lignin essentially. The percentage of lignin approximately equals K x 0.15.

Procedure:

Disintegrate the test sample (1 gm –oven-dried) in 500-ml of distilled water until free from fiber clots and undispersed fiber bundles. Avoid extensive cutting of the fibers that are separated.

Transfer the disintegrated sample to the two-litre of the beaker and add with enough distilled water to bring the total volume to 795 ml.

Adjust the temperature to 250C. Place the beaker and contents in the constant temperature bath and ensure that the temperature stays at 25 0C during the entire reaction. Continuously stir the suspension.

Pipette out 100 ml of 0.1 N $KMnO_4$ and 100 ml 4 N H_2SO_4 into a 250-ml beaker and bring this mixture to 250C. Add to it quickly the disintegrated sample and simultaneously start the stopwatch. Rinse out the 250 ml beaker, using about 5 ml of distilled water, and add to the reaction mixture. Its final volume should be 1000 ml. At the end of precisely 10.0 minutes, stop the reaction by adding 20 ml. of the 1.0 N KI from a graduated cylinder. Immediately after mixing, but without filtering out the fibers, titrate the free iodine in the suspension with 0.2 N $Na_2S_2O_3$, adding a few drops of the indicator toward the end of the reaction. Make a blank determination using exactly the same procedure but without the pulp. In this case, the mixture may be titrated with the $Na_2S_2O_3$ immediately.

Kappa number (K) was calculated as follows:

$$K=(p \times f)/w= (f \times (b-a)N)/(w \times 0.1) \qquad \text{Equation 3}$$

where

w= weight of the sample

p = amount of permanganate consumed by the sample.

f = factor for correction to a 50% permanganate consumption.

a = amount of the thiosulfate consumed by the sample.

b = amount of the thiosulfate consumed in blank titration.

N = normality of the thiosulfate

Three replicates were carried out, and the average result was reported.

3. Determination of Ash content: (TAPPI standard method: T 211 om-07)

A dried sample weighing precisely 1.0000 gm was placed in a pre-weight ceramic crucible.

This crucible was covered with a lid and heated in a muffle furnace at 550 °C±1 until complete combustion was achieved, resulting in black char.

After that, the crucible was put in a desiccator to attain room temperature.

The weight of the crucible with the ash was weighed to the nearest 0.1 mg. The ash content was calculated using equation (4)

Ash (%) = (A/ B) x 100 Equation (4)

where A is the weight of ash (g), and B is the weight of the sample (g) moisture-free

4. Determination of Lignin: (TAPPI standard method: T 222 cm-99)

In a 250 ml beaker, 1 gm of samples were taken

To it, 15 mL of 72% sulfuric acid pre-cooled to 15 ºC were added gradually. The beaker containing the sample was immersed in an ice bath to keep the temperature ≈ at 15 ºC. The sample was stirred using a glass rod to help the dispersion of samples

After that, the beaker was covered with a watch glass and kept in a bath at 20 ± 1°C for 2 hrs, with occasional stirring during this period.

The beaker's contents were then transferred slowly into a 1 L flask containing 400 mL distilled water. The beaker was rinsed with 80 mL of (3% H_2SO_4) twice, and the contents were added to the flask to ensure complete transfer of sulfuric acid and seeds for the following step.

The flask was kept under reflux with a constant volume with the help of a reflux condenser for a total period of 4 hours.

The solution was kept overnight to settle and precipitate the lignin.

Lignin was collected by filtration, dried at 105 °C to a constant weight. The percentage yield was calculated based on the original weight of the sample

5. Determination of yield (%)

The yield % of the samples was determined by using the following equation. Analysis in triplicate was carried out, and average reading was reported.

Yield %= $((W2-W3))/W1 \times 100$ Equation 5

where W1 is the mass of raw date mesh fibres

W2 is the total mass of treated fibre in a weighing container;

W3 is the mass of crucible

6. Determination of Moisture (%)

1 gm of accurately weigh samples were taken in a glass Petri dish and oven-dried at 105 °C until a constant weight was obtained. The moisture content was calculated as per the equation below. Three replicates were carried out, and the average result was reported.

$$\text{Moisture content }\% = \frac{(W_i - W_f)}{W_i} \times 100 \quad \text{Equation 6}$$

where Wi is the initial weight of the sample before drying, and wf is the weight of the sample after drying.

7. EDX analysis of the samples

Figure S1: EXD analysis of raw date palm mesh

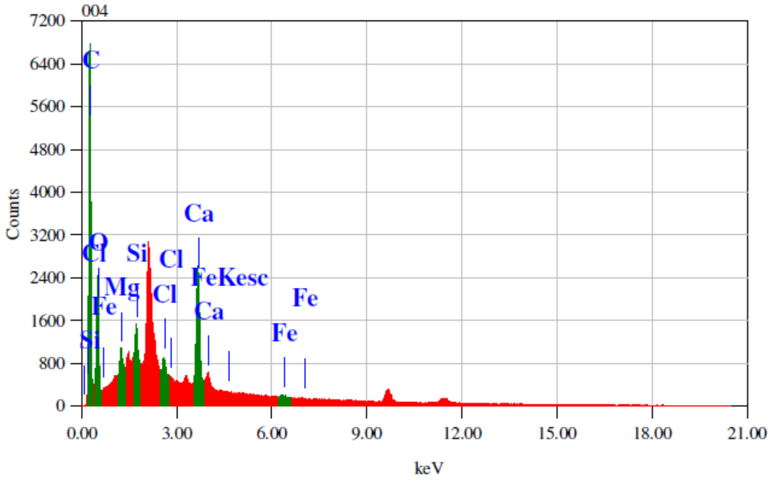


```
Acquisition Parameter
Instrument    : 6360(LA)
Acc. Voltage  : 20.0 kV
Probe Current: 1.00000 nA
PHA mode      : T3
Real Time     : 131.90 sec
Live Time     : 100.00 sec
Dead Time     : 26 %
Counting Rate: 5428 cps
Energy Range :   0 - 20 keV
```

ZAF Method Standardless Quantitative Analysis
Fitting Coefficient : 0.8869

Element	(keV)	mass%	Error%	At%	Compound	mass%	Cation	K
C K	0.277	57.50	0.49	68.93				52.8497
O K*	0.525	28.35	3.63	25.51				17.8599
Mg K*	1.253	1.14	0.86	0.68				1.3578
Si K	1.739	1.24	0.67	0.64				2.1033
Cl K*	2.621	1.29	0.77	0.52				2.8207
Ca K	3.690	9.98	1.27	3.59				22.1366
Fe K*	6.398	0.50	3.02	0.13				0.8720
Total		100.00		100.00				

Figure S2: EXD analysis of delignified mesh

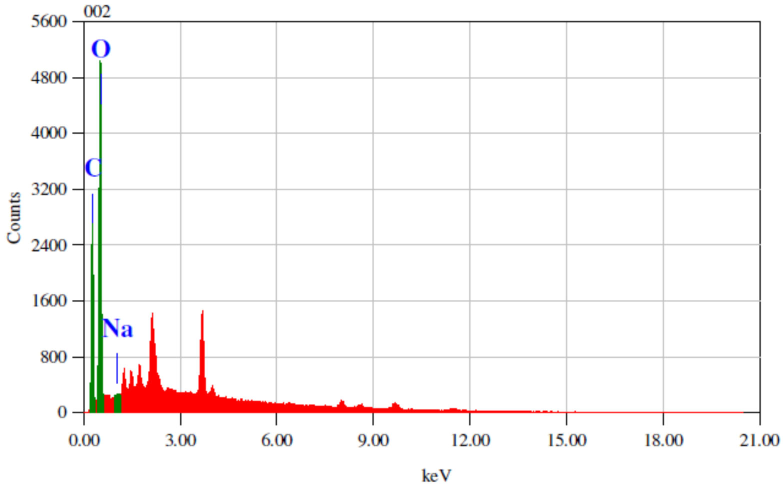

Acquisition Parameter
Instrument : 6360(LA)
Acc. Voltage : 20.0 kV
Probe Current: 1.00000 nA
PHA mode : T3
Real Time : 118.74 sec
Live Time : 100.00 sec
Dead Time : 16 %
Counting Rate: 3125 cps
Energy Range : 0 - 20 keV

ZAF Method Standardless Quantitative Analysis
Fitting Coefficient : 0.8468

Element	(keV)	mass%	Error%	At%	Compound	mass%	Cation	K
C K	0.277	39.21	0.36	46.27				32.1993
O K	0.525	60.29	1.32	53.42				67.3429
Na K*	1.041	0.50	1.11	0.31				0.4578
Total		100.00		100.00				

Figure S3: EXD analysis of cellulose

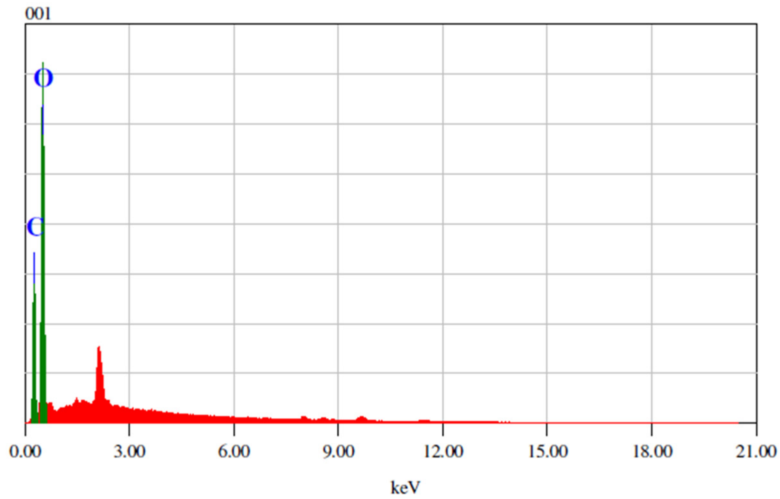

Acquisition Parameter
Instrument : 6360(LA)
Acc. Voltage : 20.0 kV
Probe Current: 1.00000 nA
PHA mode : T3
Real Time : 129.65 sec
Live Time : 100.00 sec
Dead Time : 23 %
Counting Rate: 4619 cps
Energy Range : 0 - 20 keV

ZAF Method Standardless Quantitative Analysis
Fitting Coefficient : 0.8761

Element	(keV)	mass%	Error%	At%	Compound	mass%	Cation	K
C K	0.277	38.12	0.54	45.07				30.4346
O K	0.525	61.88	1.92	54.93				69.5654
Total		100.00		100.00				

Figure S4: EXD analysis of nanocellulose

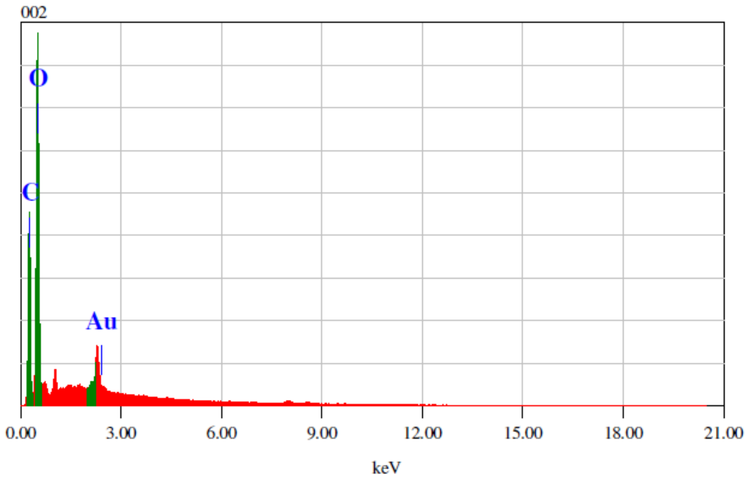

Acquisition Parameter
Instrument : 6360(LA)
Acc. Voltage : 20.0 kV
Probe Current: 1.00000 nA
PHA mode : T3
Real Time : 130.86 sec
Live Time : 100.00 sec
Dead Time : 25 %
Counting Rate: 5199 cps
Energy Range : 0 - 20 keV

ZAF Method Standardless Quantitative Analysis
Fitting Coefficient : 0.9266

Element	(keV)	mass%	Error%	At%	Compound	mass%	Cation	K
C K	0.277	41.99	0.75	49.09				37.1974
O K	0.525	58.01	3.08	50.91				62.8026
Au M*								
Total		100.00		100.00				
